# China’s product-level CO_2_ emissions dataset aligned with national input-output tables from 1997 to 2020

**DOI:** 10.1038/s41597-025-04366-5

**Published:** 2025-01-08

**Authors:** Xinbei Li, Yu Liu, Jing Zhang, Meifang Zhou, Bo Meng

**Affiliations:** 1 School of Public Policy and Management, University of Chinese Academy of Sciences, Beijing, 100049 China; 2 Institutes of Science and Development, Chinese Academy of Sciences, Beijing, 100190 China; 3 College of Urban and Environmental Sciences, Peking University, Beijing, 100871 China; 4 Institute of Carbon Neutrality, Peking University, Beijing, 100871 China; 5 China Institute of Boundary and Ocean Studies, Wuhan University, Wuhan, 430072 China; 6 College of Economics, Beijing Technology and Business University, Beijing, 100048 China; 7 Institute of Developing Economy, Japan External Trade Organization, Chiba, 2618545 Japan; 8 The Collaborative Innovation Center for Emissions Trading System Co-constructed by the Province and Ministry, Hubei University of Economics, Wuhan, 430205 China

**Keywords:** Sustainability, Environmental economics

## Abstract

Carbon emission research based on input-output tables (IOTs) has received attention, but data quality issues persist due to inconsistencies between the sectoral scopes of energy statistics and IOTs. Specifically, China’s official energy data are reported at the industry level, whereas IOTs are organized by product sectors. Valid IOT-based environmental models require consistent transformation from industry-level to product-level emissions. However, most existing studies overlook this necessary transformation, leading to substantial estimation errors. This study addresses this issue by developing a high-quality, product-level emissions dataset for China, grounded in robust product technology identification derived from IOTs. Our new emissions dataset, aligned with Chinese national IOTs, covers 29 to 34 product sectors across 7 benchmark years from 1997 to 2020. It includes data from 4 to 5 energy sectors and detailed emissions for 18 types of fossil fuels, using both IPCC-default and two China-specific emission factors. This inventory improves product-sector emission accounting and can be integrated into IOT-based climate and energy models, serving as a fundamental database for energy and emission analysis.

## Background & Summary

In light of the Paris Agreement’s clear global temperature targets, addressing climate change has assumed heightened global importance. As the world’s largest energy consumer and CO_2_ emitter^1^, China attracted significant attention in research on various emission-related issues, including product-level emission characteristics^2,3^, sector-specific mitigation strategies^4,5^, trade-related carbon transfers^6,7^, and consumption-based emission accounting^8,9^.

Sectoral carbon emission statistical accounting is fundamental to these studies. However, the inconsistency between the sectoral scopes of official energy statistics and input-output tables (IOTs) can introduce biases in the emission estimation. Models based on IOTs, such as the Environmentally Extended Input-Output (EEIO) Model^10,11^, the Computable General Equilibrium (CGE) Model^12,13^, and the Environmentally Extended social accounting matrix (ESAM)^14,15^, rely on official product-by-product IOTs to construct their databases. Notably, China’s Energy Statistics Yearbook reports energy data at the industry level, resulting in industry-level emission estimates. In contrast, China’s official IOT is presented as a product-by-product table, focusing on homogeneous product sectors rather than industry sectors. The supply table in national accounts shows that an industry produces both primary and secondary products, with secondary products represented by the non-diagonal elements in the supply table and contributing to the differences between industries and products. Therefore, to consistently construct valid models with emission accounts based on IOTs, it is essential to link product consumption with industry emissions. This requires not only aligning classifications between products and industries, but more importantly, transforming industry-level emissions into product-level emissions.

Existing studies often map emission intensity directly from industries to products without transformation^16–19^. This approach has two significant limitations. First, product-by-product IOTs capture the techno-economic linkages among product sectors in the national economy, focusing on homogeneous products. Treating industrial emission intensities as equivalent to product emission intensities undermines the accuracy of product-level studies. This discrepancy is particularly problematic in climate policy analyses, such as those involving carbon taxes, carbon emission trading, and carbon border adjustment mechanisms, where inaccurate calculations can lead to substantial biases. Second, neglecting emission transformation results in the inconsistency of indirect emissions from secondary products to their primary industry, distorting the calculation of embodied emissions and carbon footprints. For example, the power industry not only generates electricity but also engages in activities like coal mining and non-metallic mineral extraction. If calculated using industry-level emissions, the indirect emissions of secondary products such as coal and non-metallic minerals will be calculated based on the total requirements structure of power products rather than their own, leading to inconsistent and biased emission estimates.

To address the transformation of emissions from industries to products, this study employs a methodology that reallocates secondary products and derives product-by-product IOTs to estimate product-level emissions. This transformation is analogous to deriving IOTs based on supply and use tables, where the make matrix is replaced with the emissions matrix of industry sectors. The derivation of product-by-product tables relies on two basic assumptions: the product technology assumption and the industry technology assumption, which differ in handling the input structures of secondary products^20,21^. The product technology assumption (Model A) posits that each product is produced in its specific means of production, regardless of the industry where it is produced. In other words, different industries use the same input structure to produce the same product. In contrast, the industry technology assumption (Model B) assumes that each industry applies a unique production process to all products it produces, regardless of the product mix. Theoretically, the product technology assumption is more applicable in cases of secondary production and performs better in deriving product-by-product IOTs^20,22,23^, while the industry technology assumption is proved to violate the input-output rules of financial balance, price invariance, and scale invariance^24,25^. In product-level emission accounting, the product technology assumption is also more realistic. For example, the industry technology assumption would suggest that steel and coke produced by the iron and steel industry emit the same CO_2_ per unit of output, which is clearly not the case.

To improve carbon emission accounting at the product-sector level, this study employs the product technology assumption to convert industry-level emissions into product-level emissions. The dataset estimates CO_2_ emissions for 29 to 34 product sectors in China across 7 benchmark years (1997, 2002, 2007, 2012, 2017, 2018, and 2020), covering 18 specific fossil fuels (see Table 1) and 4 to 5 energy sectors consistent with IOTs (see Supplementary Table S2). Furthermore, the dataset provides emission estimates based on both Intergovernmental Panel on Climate Change (IPCC) default emission factors and two sets of China-specific emission factors, offering more detailed data to support future research. This study advances our previous work^26^ in in three key areas: emission factor uncertainty, fossil fuel aggregation bias, and the inclusion of long-time series data. The necessity of emission transformation from industries to products is validated through a comparison of emissions at both levels, and an uncertainty analysis is conducted using Monte Carlo simulations.

**Table 1:** Fossil fuels and emission factors.

No.	Fuels in this study	Fuels in China’s Energy Statistics	${{\boldsymbol{EF}}}_{{\boldsymbol{k}}}^{{\boldsymbol{IPCC}}}$ (Kg CO_2_/TJ)	${{\boldsymbol{EF}}}_{{\boldsymbol{k}}}^{{\boldsymbol{CEADs}}}$ (Kg CO_2_/TJ)	${{\boldsymbol{EF}}}_{{\boldsymbol{k}}}^{{\boldsymbol{NDRC}}}$ (Kg CO_2_/TJ)
1	Raw coal	Raw coal	97,967	96,500	96,690
2	Cleaned coal	Cleaned coal	97,967	96,500	93,170
3	Other washed coal	Other washed coal	97,967	96,500	93,170
4	Briquettes	Briquettes	97,500	96,500	123,053
		Gangue	97,967	96,500	123,053
5	Coke	Coke	107,000	115,060	107,900
6	Coke oven gas	Coke oven gas	44,400	78,800	49,800
7	Other gas	Blast furnace gas, Converter gas, Other gas	260,000	78,800	44,700
8	Other coking products	Other coking products	97,500	100,650	108,200
9	Crude oil	Crude oil	73,300	73,627	73,627
10	Gasoline	Gasoline	70,000	69,300	69,300
11	Kerosene	Kerosene	71,900	71,900	71,900
12	Diesel oil	Diesel oil	74,100	74,100	74,100
13	Fuel oil	Fuel oil	77,400	77,400	77,400
14	Liquefied Petroleum Gas	Liquefied Petroleum Gas	63,100	73,300	63,100
15	Refinery gas	Refinery Gas	57,600	74,100	66,700
16	Other petroleum products	Naphtha, Lubricants, Petroleum waxes, White spirit, Other petroleum products	73,300	63,100	73,300
		Bitumen asphalt	80,700	63,100	80,700
		Petroleum coke	97,500	63,100	100,800
17	Natural gas	Natural gas	56,100	56,173	56,173
18	Liquefied Natural Gas	Liquefied Natural Gas	64,200	64,200	63,100

### Data source

All sectoral energy and input-output data used in this paper are sourced from publicly available official agencies and academic institutions. Specifically, China’s energy balance, final energy consumption by industrial sector, and average low calorific value of fossil fuels are obtained from the *China Energy Statistics Yearbook*^38^, compiled by the National Bureau of Statistics (NBS). The supply tables are derived from the *Input-Output Tables of China*^39^, which are updated every five years. Since 1997, China has officially published supply tables for only seven years, including extended tables for two of those years. Due to data limitations, secondary product data for the wholesale and retail, transportation and postal, and other service sectors were unavailable from 1997 to 2012. To maintain consistency, we adjusted the supply data for earlier years based on the 2017 data. Emissions factors used in this study are derived from the *2006 IPCC Guidelines for National Greenhouse Gas Inventories*^27^, the CEADs developed by Liu *et al*.^28^ and Shan *et al*.^29^, and the NDRC’s *Guidelines for Provincial Greenhouse Gas Inventories (Trial)*^30^.

## Methods

### Product/industry boundaries and accounting scopes

The concepts of industry and product in this study align with the definitions in the *2008 System of National Accounts*^21^ (2008 SNA). According to the 2008 SNA, an industry is defined as a group of establishments engaged in the same or similar types of production, while products are the goods and services resulting from these production processes. It is important to note that there is not always a one-to-one correspondence between production activities and products, and consequently, between industries and products. For instance, some activities simultaneously produce multiple products, such as beef and hides obtained from animal slaughter, and the same product may be produced by different activities, such as cheese produced by either the dairy industry or farms. In practice, most industries engage in both principal and secondary activities, further widening the discrepancy between industry and product. According to supply tables, the secondary output in China averages between 1.6% and 6.5% of total output from 1997 to 2020. However, the level of secondary activities varies significantly across sectors. For example, the general level of secondary activities in China’s industries was 3.1% in 2020, whereas it reached 12.5% in the carbon-intensive industry “Processing of Petroleum, Coal, and Other Fuels”. The product-by-product IOTs are classified by products with high homogeneity in terms of cost structures and production activities, making the transformation of emissions from industries to products essential for accurate input-output analysis.

**Fig. 1:**
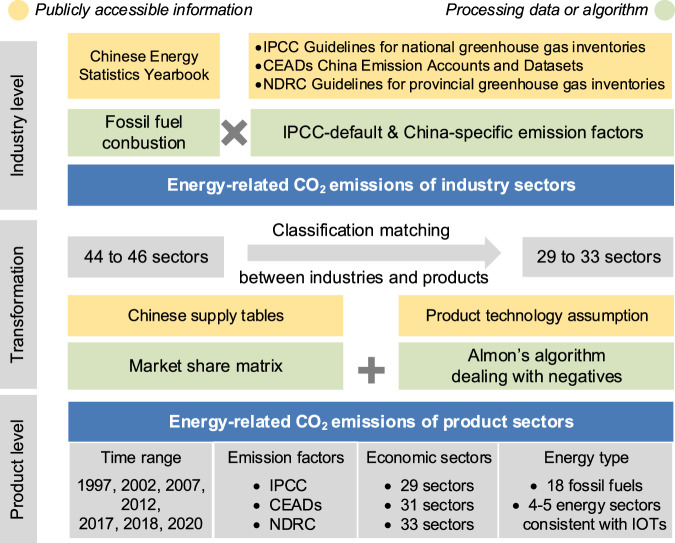
Framework of China’s emission accounting for product sectors based on product technology assumption.

The CO_2_ emissions in this dataset are estimated according to the administrative territorial-based accounting scopes defined by IPCC. Moreover, the emission inventory includes only CO_2_ emissions resulting from fossil fuel combustion, specifically energy-related emissions. The process of emissions accounting for product sectors based on product technology assumption is illustrated in Fig. 1.

### Energy-related industrial CO_2_ emissions accounting

The energy-related CO_2_ emissions of industries are calculated based on industrial energy combustion, as shown in Eq. 1.

1 $${C}_{{jk}}={E}_{{jk}}\times {{CV}}_{k}\times {EF}_{k}\times {O}_{{jk}}$$

where ${C}_{{jk}}$ represents the CO_2_ emissions from burning the fossil fuel *k* in the *j*-th industry, ${E}_{{jk}}$ represents the physical quantity of fuel *k* combusted in the *j*-th industry, ${{CV}}_{k}$ represents the average low calorific value produced by per physical unit of fuel *k*, ${{EF}}_{k}$ refers to the CO_2_ emissions per unit of caloric value produced by fuel *k*, and ${O}_{{jk}}$ refers to oxygenation efficiency during fuel *k* combustion in the *j*-th industry. Most existing research on China’s emission estimation relies on the IPCC default emission factors^27^ (${{EF}}_{k}^{{IPCC}}$). However, both China Emission Accounts and Datasets (CEADs)^28,29^ and National Development and Reform Commission of China (NDRC)^30^ have published China-specific values (${{EF}}_{k}^{{CEADs}}$, ${{EF}}_{k}^{{NDRC}}$), which differ significantly from the IPCC defaults. Liu *et al*.^28^ found that the surveyed emission factors of China’s coal are approximately 40% lower than those recommended by IPCC. In light of these differences, this study employs both the IPCC default emission factors and two sets of China-specific factors to estimate the industry-level emissions in China, as detailed in Table 1.

The key to accurately calculating the energy consumption from combustion is to deduct the non-combustion energy input from the total energy consumption. According to the China’s energy balance, energy combustion includes final energy consumption, and inputs for thermal power and heating supply, excluding those used as raw materials (classified as non-energy use). We adopted the method proposed by Peters *et al*.^31^ to account for non-energy use in various sectors. It is assumed that non-energy use is concentrated in the chemical industry. And if there is any surplus after deducting chemical raw materials, it will be distributed equally among non-chemical industries. The calculation for industrial energy combustion is shown in Eq. 2.

2 $${E}_{{jk}}=\left\{\begin{array}{ccc}{E}_{{jk}}^{T}+{E}_{k}^{P}+{E}_{k}^{H}-{E}_{{jk}}^{M}, & {if} & j\in {type}1\\ {E}_{{jk}}^{T}-{E}_{{jk}}^{M}, & {if} & j\in {type}2\\ {E}_{{jk}}^{T}, & {if} & j\in {type}3\end{array}\right.$$

where ${E}_{{jk}}^{T}$ represents the final consumption of fossil fuel *k* by the *j*-th industry, ${E}_{k}^{P}$ represents the consumption of fossil fuel *k* for thermal power generation, ${E}_{k}^{H}$ represents the consumption of fuel *k* for heating supply, and ${E}_{{jk}}^{M}$ represents the use of fuel *k* as non-energy use in the *j*-th industry. Industry type 1 refers to the production and distribution of electric power and heat power. Industry type 2 includes the mining industry, the manufacturing industry, the production and distribution of gas and water, construction and transportation. The remaining sectors are classified as industry type 3. It is important to note that China’s official institutions only provide statistics on non-energy use in industry. However, other petroleum products, such as petroleum asphalt and lubricants, are usually used for road paving, roofing and lubrication in the transportation and construction sectors, without contributing to fuel combustion emissions. In line with the IPCC methodology^27^, these inputs of other petroleum product in the transportation and construction sectors are classified as non-energy products.

To enhance data transparency and verifiability, we provide China’s energy inventory for each industry sector in the dataset (see file “China industrial energy inventory, 1997 to 2020”). Researchers can use the provided Matlab codes^32^ and energy inventories to recalculate industry-level CO_2_ emissions with different emission factors and convert them to product-level emissions.

### Classification matching between industries and products

Using the above method, the combustion emissions for 46 (or 44) industries and 18 fossil fuels were obtained. To link the product production with industry emissions, it is necessary to manually match the classifications between industries in the energy statistics and those in the supply tables. To retain as many sectors as possible, the industries were merged into 29 sectors in 1997, 2002 and 2007, 31 sectors in 2012, and 33 sectors in 2017, 2018 and 2020. Detailed sector matching is provided in Supplementary Table S3.

### Emissions transformation from industries to products

The product technology assumption supposes that the production methods and input structure for a given product are identical, regardless of the industry in which it is produced. This implies that the CO_2_ emission intensity of a product is assumed to be the same regardless of the industry making it. Consequently, the emissions of an industry sector can be considered a weighted average of the emissions from each product, with the weights corresponding to the market share of each product manufactured by that industry sector, as shown in Eq. 3 to Eq. 5.

3 $${d}_{{ij}}=\frac{{s}_{{ij}}}{{x}_{i}}$$

4 $${\boldsymbol{C}}={\boldsymbol{P}}\times {\boldsymbol{D}}$$

5 $$\left[\begin{array}{ccc}{C}_{11} & \cdots  & {C}_{1j}\\ \vdots  & \ddots  & \vdots \\ {C}_{k1} & \cdots  & {C}_{{kj}}\end{array}\right]=\left[\begin{array}{ccc}{P}_{11} & \cdots  & {P}_{1i}\\ \vdots  & \ddots  & \vdots \\ {P}_{k1} & \cdots  & {P}_{{ki}}\end{array}\right]\left[\begin{array}{ccc}{d}_{11} & \cdots  & {d}_{1j}\\ \vdots  & \ddots  & \vdots \\ {d}_{i1} & \cdots  & {d}_{{ij}}\end{array}\right]$$

where ${s}_{{ij}}$ denotes the output of the *i*-th product produced by the *j*-th industry, and ${x}_{i}$ denotes the total output of the *i*-th product, with the origin data derived from the China supply table. ${d}_{{ij}}$ indicates the share of the *i*-th product produced by the *j*-th industry, that is, the market share. ***D*** denotes the market shares matrix. ${\boldsymbol{C}}$ represents the CO_2_ emission matrix of industry sectors, with the element ${C}_{{kj}}$ denoting the CO_2_ emission generated by burning fuel *k* in the *j*-th industry sector. ${\boldsymbol{P}}$ represents the CO_2_ emission matrix of product sectors, with the element ${P}_{{ki}}$ denoting the CO_2_ emission generated by combustion of fossil fuel *k* in the *i*-th product.

Therefore, the CO_2_ emissions of product sectors are estimated as shown in Eq. 6.

6 $${\boldsymbol{P}}={\boldsymbol{C}}\times {{\boldsymbol{D}}}^{-1}$$

It is important to note that the product technology assumption may result in negative values in the solution. This issue arises in the inverse matrix of the market share coefficient matrix (${{\boldsymbol{D}}}^{-1}$), which can lead to negative values in the emission matrix of product sectors (${\boldsymbol{P}}$) under the product technology assumption. These negatives are primarily attributable to factors such as ancillary activities accounting, heterogeneity in data and classifications, and statistical errors^20,33^. Several methods have been developed to address this issue, including matrix multiplication^20^, Almon’s algorithm^34^, and the activity technology model^35^. Among these, Almon’s algorithm is considered the most widely accepted and compatible with product technology assumption^36,37^. It is an iterative, product-technology based algorithm that introduces the scale factors to scale down the removal and entry terms of secondary products during the iteration process and automatically eliminate the negatives row by row. The derivation process and formulas for Almon’s algorithm are detailed in the Supplementary Text S1. This method is recommended in the *Eurostat Manual of Supply, Use and Input-Output Tables*^20^, and has been extensively used by the European Union countries to derive non-negative product-by-product IOTs.

We employ the product technology assumption and Almon’s method to eliminate the resulting negatives and transform industry-level emissions into product-level emissions. To align with studies using IOT-based models, 18 fossil fuels are aggregated into energy sectors consistent with China’s official IOTs, with classification matching details provided in Supplementary Table S2. Additionally, we have made the Almon’s procedure available as a Matlab script on the Figshare^32^, which allows researchers to execute this process without requiring a deep understanding of the underlying formulas or any additional programming.

## Data Records

The dataset generated in this study is accessible at the Figshare^32^, and all the data are compiled in the XLSX format. The dataset includes the following data, covering seven benchmark years (1997, 2002, 2007, 2012, 2017, 2018 and 2020):

1. A total of 11,718 CO_2_ emissions records for 29-34 product sectors and 18 types of fossil fuels across the seven benchmark years, estimated using IPCC default emission factors and two sets of China-specific values from CEADs and NDRC [file “China product-level emission inventory by fossil fuels, 1997 to 2020”];
2. A total of 2,901 CO_2_ emissions records for 29-34 product sectors and 4-5 IOT energy sectors across the seven benchmark years, estimated using IPCC default emission factors and two sets of China-specific values from CEADs and NDRC [file “China product-level emission inventory by energy sectors, 1997 to 2020”];
3. A total of 4,989 energy records for 29-34 product sectors and 18-27 types of fossil fuels across the seven benchmark years [file “China industrial energy inventory, 1997 to 2020”].

## Technical Validation

### Comparison with industry-level emissions

**Fig. 2:**
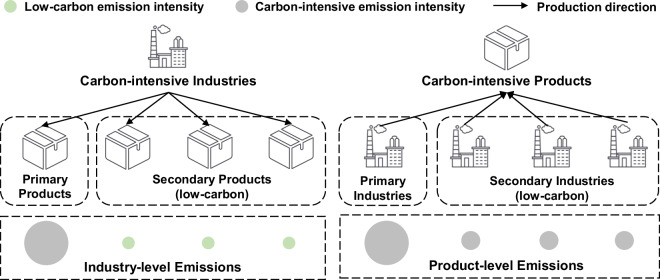
Conceptual diagram of differences between industry-level and product-level emissions in carbon-intensive sectors. The color of the circles denotes emission intensity, while the size reflects the volume of emissions. The discrepancy between industry-level and product-level emissions is illustrated by the difference between the green circles on the left and the corresponding gray circles on the right.

A substantial disparity exists between product-level and industry-level emissions after transformation (see Supplementary Table S4), underscoring the importance of accurate emission transformation from industry to product in related studies. For homogeneous sectors without secondary products, such as agriculture and construction, the scope of industry-level emissions in energy statistics aligns with that the product-sector scope in the IOTs, resulting in consistent emissions estimates across both levels. However, for sectors with secondary products, industry-level emissions tend to underestimate CO_2_ emissions from high-emitting sectors (e.g. production and supply of power and heat sector), and overestimate emissions from medium- and low-emitting sectors (e.g. manufacture of metal products, and mining and washing of coal), when compared to product-level emissions. Figure 2 illustrates the source of accounting bias between industry-level and product-level emissions in carbon-intensive sectors, primarily arising from input-output linkages and emission disparities among production-linked sectors. Specifically, industry-level emissions include those from low-carbon products manufactured by carbon-intensive industries, whereas product-level emissions capture emissions from carbon-intensive products generated by secondary industries. This leads to an underestimation of emissions in carbon-intensive sectors, while emissions in low-carbon sectors are typically overestimated at the industry level. The gap between industry-level and product-level emissions is more pronounced when the emissions gap between primary and secondary products is larger, and when the market share of secondary products is higher. A detailed analysis of the deviations between industry-level and product-level emissions can be found in our previous study^26^ and Supplementary Text S2.

For sectors that produce secondary products, the largest disparity between industry-level and product-level emissions occurs in the production and supply of electricity and heat. From 2007 to 2020, the discrepancies consistently exceeded 100 Mt CO_2_, where emissions are underestimated by 158.5-168 Mt in 2017 and 159-169 Mt in 2018. These discrepancies are greater than the total CO_2_ emissions of the Netherlands in those years, which were 156 Mt and 151 Mt, respectively (World Bank data: https://data.worldbank.org/indicator/EN.ATM.CO2E.KT). In terms of deviation rate, among sectors with emissions exceeding 10 Mt CO_2_, the mining and washing of coal sector exhibits the highest deviation, with an average discrepancy rate of 78%-84% from 1997 to 2020 (using product-level emissions as the baseline).

### Comparison with emissions transformation based on industry technology assumption

Although the product technology assumption is theoretically robust for constructing product-by-product IOTs, the industry technology assumption offers the advantage of avoiding negative values. To assess the empirical implications of the industry technology assumption for emissions transformation, we estimate China’s product-level emissions for seven benchmark years using the industry technology assumption (see Supplementary Table S5). The results reveal significant discrepancies between product-level emissions estimated using the industry and product technology assumptions. Notably, for 81.5%-93.5% of sectors with secondary products, the deviations between product-level emissions under the industry and product technology assumptions exceed those between industry-level and product-level emissions using the product technology assumption. For example, the largest difference occurs in the production and supply of electricity and heat sector, where emissions are underestimated by 202 Mt CO_2_ in 2017. The mining and washing of coal sector shows the greatest difference rate, with an average deviation of 101%-112% from 1997 to 2020 (using product-level emissions as the baseline). A detailed formulaic explanation of why the industry technology assumption leads to larger deviations is provided in Supplementary Text S2.

### Comparison with existing datasets

Most publicly available databases provide only total emissions for China, with a few offering emission datasets for the country’s economic sector. Notable examples include CEADs (https://www.ceads.net/data/nation?#284), the World Input-output Database (WIOD, https://www.rug.nl/ggdc/valuechain/wiod/wiod-2016-release), Eora national environmental satellite accounts (Eora, https://worldmrio.com/countrywise/), and EXIOBASE3 (https://zenodo.org/record/5589597). Among these, CEADs and WIOD provide the industry-level emissions, while EXIOBASE3 and Eora offer product-level emissions aligned with global multi-regional input-output tables. EXIOBASE3 estimates emissions using the energy supply and use data from the International Energy Agency (IEA), converting these into product-level emissions based on supply-use tables and industry technology assumption^40^. In contrast, Eora offers detailed sectoral emissions from fuel combustion, derived from the United Nations Framework Convention on Climate Change (UNFCCC) and IEA data^41,42^. However, there is no evidence to support the emission transformation used in Eora’s satellite accounts.

**Fig. 3:**
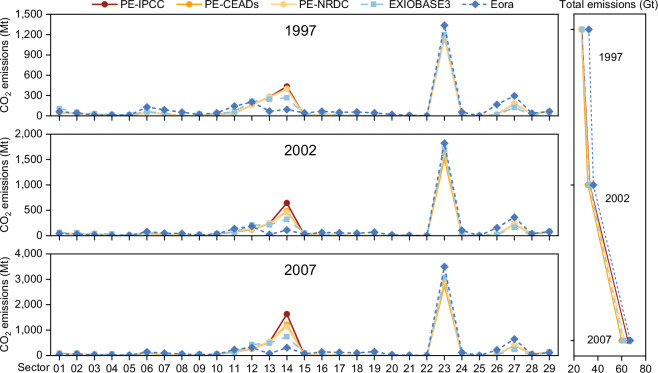
Comparisons with other product-level emissions in 1997, 2002 and 2007. PE-IPCC, PE-CEADs and PE-NDRC represent the product-level emissions based on the IPCC emission factor, and China-specific emission factors from CEADs and NDRC, respectively. The sector names corresponding to the sector numbers can be found in the Supplementary Table S3.

Despite these differences, comparisons between our dataset and other product-level emissions databases remain essential for technical validation. Given that EXIOBASE3, Eora, and our dataset cover different time periods—EXIOBASE3 covers 1995–2011, the free version of Eora covers 1990–2016, and our dataset spans seven benchmark years during 1997-2020—we selected three overlapping years (1997, 2002, and 2007) for comparison. To facilitate this, we aggregated the product sectors in EXIOBASE3 and Eora to align with our dataset’s sectoral classification of 29 sectors. The results in Fig. 3 reveal a general agreement in product-level emissions among our dataset, EXIOBASE3, and Eora. However, Eora tends to overestimate the total emissions, while significantly underestimating emissions in sectors such as manufacture of non-metallic mineral products (Sector 13) and smelting and pressing of metals (Sector 14). This discrepancy is likely due to differences in source data and emission allocation methods, as Eora relies on data from the UNFCCC and IEA^43,44^.

Compared to other databases, our dataset offers contributions by employing the product technology assumption, which aligns with more accurately China’s product-by-product IOTs. It also fully incorporates China-specific energy statistics, emission factors, and supply tables. Furthermore, the most recent available data in Eora and EXIOBASE3 are from 2016 and 2011, and our dataset extends to 2020. Therefore, our work provides a more accurate, comprehensive, and up-to-date product-level carbon emissions inventory aligned with China’s official energy statistics and IOTs, serving as a significant supplement to existing emission estimates.

### Uncertainty analysis

**Fig. 4:**
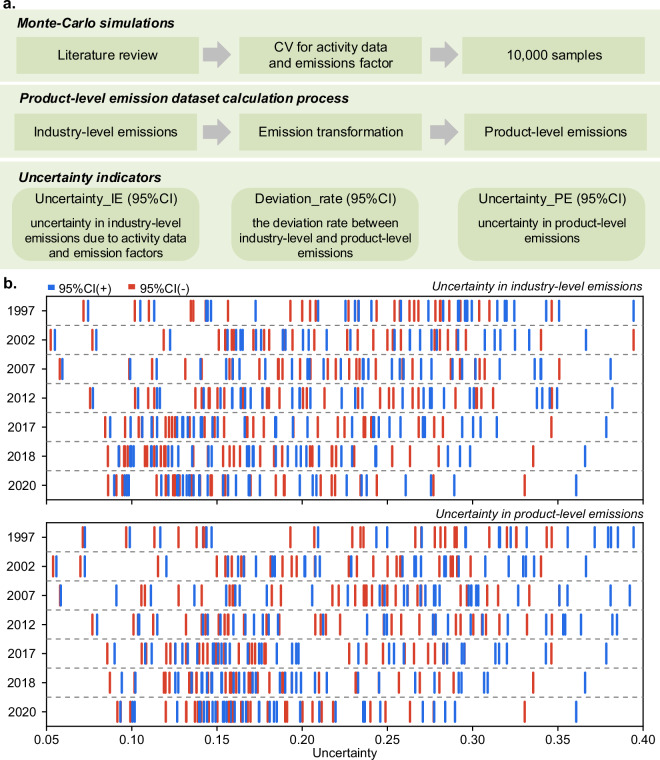
Uncertainty of product-level emission datasets. (**a**) The framework of uncertainty analysis. (**b**) The uncertainty ranges in industry-level and product-level emissions from 1997 to 2020. The blue and red whiskers represent the upper and lower limit of sectoral uncertainty within a 95% confidence interval (95% CI).

Uncertainty analysis is essential for improving the accuracy of emission inventories. According to the IPCC, uncertainty in sectoral CO_2_ emission inventories mainly stems from modeling, activity data, and emission factors. In this study, emissions are first estimated at the industry level, and then converted into product-level emissions. The uncertainty at the industry level primarily arises from the variability in activity data and emission factors, which are carried over during the emissions transformation process. Furthermore, this transformation introduces deviations between industry-level and product-level emissions. To address these uncertainties, we developed a framework that accounts for both industry-level and product-level uncertainties, as shown in Fig. 4a. Based on this framework, three indicators for uncertainty analysis are estimated in Supplementary Table S6.

The activity data used in this study are sourced from the *Energy Statistics Yearbook*, compiled by China’s NBS. While these data are officially published, they are frequently subject to revisions^45,46^. For instance, Korsbakken *et al*.^47^ noted that revisions following the Third National Economic Census led to an 11% increase in China’s carbon emissions for 2013. Although this study incorporates the most recent energy statistics, including the latest official revisions from the NBS, uncertainties persist due to the non-transparent data collection, reporting, and validation within China’s statistical system^48^. Shan *et al*.^49^ reported the coefficient of variation (CV) for China’s energy statistics varies across sectors, ranging from 5% in the power generation sector, 10% in the industry and building sector, 16% in the transportation sector, and to as high as 30% in the agricultural sector.

Regarding energy emission factors, this study uses those published by the IPCC, CEADs and NDRC. However, several sources of energy emission factors are available in China, with significant discrepancies observed across energy types. Referring to Shan *et al*.^29^, a comparison of eight different emission factors reveals a CV ranging from 1.1% (crude oil) to 35.4% (other gases).

To quantify uncertainty in sectoral emissions estimates under the product technology assumption, we employed the Monte-Carlo method, as recommended by the IPCC. Assuming that industrial activity data and emission factors follow a normal distribution, we generated 10,000 random samples for each CV to produce 10,000 sets of sectoral CO_2_ emission estimates. The uncertainty of industry-level emissions for 1997–2020 is illustrated in Fig. 4b, revealing that uncertainty is concentrated between 7% and 35% within a 95% confidence interval. Notably, higher uncertainty in activity data leads to the agriculture sector exhibiting the highest emissions uncertainty, ranging from −33% to 39%. In contrast, the petroleum and natural gas extraction sector shows the lowest emissions uncertainty, ranging from -5% to 9%.

After converting industry-level emissions to product-level emissions using product technology assumptions, we observe notable deviations, which vary across sectors. In sectors without secondary products, such as agriculture and construction, the deviation is zero. However, sectors like mining and washing of coal, textiles, and smelting and pressing of metals, exhibit deviations significantly greater than the uncertainty associated with activity data and emission factors. For instance, the average deviation rate in the mining and washing of coal sector ranges from 53% to 98%, far exceeding the average uncertainty in industry-level emissions (from -24% to 25%). Conversely, sectors like food manufacturing and non-metallic mineral products show smaller deviations. In food manufacturing, the average deviation rate ranges from 5% to 9%, while the average uncertainty in industry-level emissions spans -26% to 29%.

Overall, 37% to 52% of sectors with secondary products have deviation rates that surpass the uncertainties related to activity data and emission factors, emphasizing the need to address these discrepancies when estimating product-level emissions.

## Usage Notes

The emissions transformation method recommended in this paper requires ensuring consistency between the industry emission database and the supply tables to ensure that the program performs correctly when converting the emissions. Additionally, convergence of the Almon’s algorithm can only be guaranteed if more than half of each product is produced by its primary industry in the supply tables^34^. The algorithm is effective for addressing smaller negative values; however, larger negatives may indicate errors in the source data, and it is preferable to correct these source values rather than relying on the Almon’s program to automatically correct them^20,36^.

## Supplementary information

Supplementary Tables

Supplementary Information

## Data Availability

The emission transformation from industries to products and Monte-Carlo simulation results processing were performed using Matlab 2016a, while other calculations were completed in Excel. The editable Matlab scripts are available on the open-access online database Figshare (10.6084/m9.figshare.26928094). Although the provided Almon’s transformation procedure is specifically designed for emissions transformation, it can also be applied to derive the product-by-product IOTs by substituting the use tables for the industry-level emission matrix.
