# Supplementary material for: China’s product-level CO2 emissions dataset aligned with national input-output tables from 1997 to 2020
Source: Sci Data. 2025 Jan 8;12:30. doi: 10.1038/s41597-025-04366-5 (PMC11711322; doi:10.1038/s41597-025-04366-5)
Supplement: Supplementary file 2 — Supplementary Information [file 41597_2025_4366_MOESM2_ESM.docx]

**Supplementary Information of**

**China’s Product-Level CO_2_ Emissions Dataset Aligned with National Input-Output Tables from 1997 to 2020**

Xinbei Li^3,4^, Yu Liu^1,2*^, Jing Zhang^5^, MeifangZhou^6^, Bo Meng^7,8^

1. College of Urban and Environmental Sciences, Peking University, Beijing 100871, China

2. Institute of Carbon Neutrality, Peking University, Beijing 100871, China

3. School of Public Policy and Management, University of Chinese Academy of Sciences, Beijing 100049, China

4. Institutes of Science and Development, Chinese Academy of Sciences, Beijing 100190, China

5. China Institute of Boundary and Ocean Studies, Wuhan University, Wuhan 430072, China

6. College of Economics, Beijing Technology and Business University, Beijing 100048, China

7. Institute of Developing Economy, Japan External Trade Organization, Chiba 2618545, Japan

8. The Collaborative Innovation Center for Emissions Trading System Co-constructed by the Province and Ministry, Hubei University of Economics, Wuhan 430205, China

*corresponding author: Yu Liu (yu.liu@pku.edu.cn)

**Contents of this file**

**Text S1.** Derivation and formulas of Almon’s algorithm.

**Table S1.1.** A simplified use table.

**Table S1.2.** A simplified supply table.

**Table S1.3.** A simplified input-output table.

**Text S2.** Derivation of deviations between emissions estimated by product technology assumption and other methods.

**Table S2.** Classification matching between fossil fuels and energy sectors.

**Text S1. Derivation and formulas of Almon’s algorithm.**

Almon^1^ developed an iterative, product-technology based algorithm that uses the scale factors to automatically eliminate the negatives, calculating the IOTs row by row and addressing negatives as soon as they appear. This method is recommended in the *Eurostat Manual of Supply, Use and Input-Output Tables*^2^, and has been extensively used by the European Union countries to derive non-negative product-by-product IOTs. The derivation and formulas are as follows.

Table S1.1 to Table S1.3 present the simplified supply, use and input-output tables for China. The supply table outlines the origins of goods and services by industry, while the use table details their destinations by type of use within the economy.

**Table S1.1.** A simplified use table.

| Industries  Products | | Immediate use | | | | Final demand | Total |
| --- | --- | --- | --- | --- | --- | --- | --- |
|  |  | 1 | 2 | … | *j* |  |  |
| Immediate inputs | 1 | $\boldsymbol{U}=\left( u_{ij} \right)$ | | | | ***F*** | $\boldsymbol{X}$ |
|  | 2 |  |  |  |  |  |  |
|  | … |  |  |  |  |  |  |
|  | *i* |  |  |  |  |  |  |
| Value added | | $\boldsymbol{V}^{\boldsymbol{I}}$ | | | |  |  |
| Total | | $\boldsymbol{X}^{\boldsymbol{I}}$ | | | |  |  |

**Table S1.2.** A simplified supply table.

|  | | Industries | | | | Total |
| --- | --- | --- | --- | --- | --- | --- |
|  |  | 1 | 2 | … | *j* |  |
| Products | 1 | $\boldsymbol{S=}\left( \boldsymbol{s}_{\boldsymbol{ij}} \right)$ | | | | ***X*** |
|  | 2 |  |  |  |  |  |
|  | … |  |  |  |  |  |
|  | *i* |  |  |  |  |  |
| Total | | $\boldsymbol{X}^{\boldsymbol{I}}$ | | | |  |

**Table S1.3**. A **s**implified input-output table.

| Products  Products | | Immediate use | | | | Final demand | Total |
| --- | --- | --- | --- | --- | --- | --- | --- |
|  |  | 1 | 2 | … | *j* |  |  |
| Immediate inputs | 1 | $\boldsymbol{Z=}\left( \boldsymbol{z}_{\boldsymbol{ij}} \right)$ | | | | ***F*** | ***X*** |
|  | 2 |  |  |  |  |  |  |
|  | … |  |  |  |  |  |  |
|  | *i* |  |  |  |  |  |  |
| Value added | | ***V*** | | | |  |  |
| Total | | ***X*** | | | |  |  |

Based on the equilibrium between the total use of product sectors in the use table, the corresponding equations can be established, as shown in Eq. S1.

| $\boldsymbol{X}=\boldsymbol{U}+\boldsymbol{F}=\boldsymbol{H}\boldsymbol{X}^{I}+\boldsymbol{F}$ | (S1) |
| --- | --- |

where ***F*** and ***X*** denote the final use matrix and the total use (output) column vector of the product sectors, respectively. $\boldsymbol{U}$ denotes the use matrix of the use table, with element $u_{ij}$ representing the value of the *i*-th product consumed by the *j*-th industry sector. $\boldsymbol{X}^{\boldsymbol{I}}$ is the row vector of total inputs of the industry sectors. ***H*** represents the input coefficients matrix of the use table, where element $h_{ij}$ indicates the value of the *i*-th product consumed per unit of output value in the *j*-th industry.

The product mix matrix in the supply table can be expressed as:

| $\boldsymbol{T}=\boldsymbol{S}\times\left( \hat{\boldsymbol{X}^{I}} \right)^{-1}$ | (S2) |
| --- | --- |

where $\boldsymbol{S}$ denotes the supply matrix of the supply table, with element $s_{ij}$ indicating the output of the *i*-th product produced by the *j*-th industry. $\boldsymbol{T}$ denotes the product mix matrix, with element $t_{ij}$ representing the fraction of the total output of the *j*-th industry that is in the form of *i*-th product. $\hat{\boldsymbol{X}^{\boldsymbol{I}}}$ denotes the diagonal matrix of the total output row vector $\boldsymbol{X}^{\boldsymbol{I}}$ of the industries.

When ***T*** is full rank, the Eq. S3 for the total output and final demand of products under the product technology assumption can be derived based on Eq. S1-S2.

| $\boldsymbol{X}=\left( \boldsymbol{I}-\boldsymbol{H}\boldsymbol{T}^{-1} \right)^{-1}\boldsymbol{F}$ | (S3) |
| --- | --- |
| ${\boldsymbol{X}=\left( \boldsymbol{I}-\mathbf{A} \right)}^{-1}\boldsymbol{F}$ | (S4) |

where ***A*** denotes the direct input coefficient matrix, with element $a_{ij}$ representing the inputs of *i*-th product directly consumed by the *j*-th product sector. According to the Leontief model (Eq.S4), we can derive that $A=HT^{-1}$, then $H=AT$. This indicates the input coefficient of a product to an industry is the weighted average of direct input coefficients of that product to each of the product that the industry makes, and the weights are the shares of each product in that industry’s total output (i.e. the product mix coefficient).

Therefore, the matrix of direct product inputs for product output under the product technology assumption can be derived as follows.

| $\boldsymbol{Z}=\boldsymbol{A}\hat{\boldsymbol{X}}=\boldsymbol{HT}^{-1}\hat{\boldsymbol{X}^{I}}=\boldsymbol{U}\left( \hat{\boldsymbol{X}^{I}} \right)^{-1}\times\boldsymbol{S}^{-1}\left( \hat{\boldsymbol{X}^{I}} \right)\times\hat{\boldsymbol{X}}=\boldsymbol{U}\boldsymbol{S}^{\mathbf{-1}}\hat{\boldsymbol{X}}$ | (S5) |
| --- | --- |

where ***Z*** denotes the inter-sector transaction matrix, with element $z_{ij}$ representing the *i*-th product direct inputs for the production of the *j*-th product. $\hat{\boldsymbol{X}}$ denotes the dominant diagonal matrix of the product outputs. ***D*** represents the market share matrix from the supply table, with element denoting the fraction of total product *i* output produced by the *j*-th industry.

Based on the Eq. S6 which calculates the market share matrix, the inter-sector transaction matrix ***Z*** can be derived using the make matrix ***U*** fromthe use table and the market share matrix ***D*** from the supply table.

| $\boldsymbol{D}=\boldsymbol{S}\times{\hat{\boldsymbol{X}}}^{-1}$ | (S6) |
| --- | --- |
| $\boldsymbol{Z}=\boldsymbol{U}\boldsymbol{D}^{-1}$ | (S7) |

According to the Eq. S7, Almon’s method constructed the iterative formulas:

| $\boldsymbol{Z}=\boldsymbol{U}+\boldsymbol{Z}\left( \boldsymbol{I}-\boldsymbol{D} \right)$ | (S8) |
| --- | --- |

Starting with $Z^{(0)}=U$, we then define successive approximations as follow.

| $\boldsymbol{Z}^{(k+1)}=\boldsymbol{U}+\boldsymbol{Z}^{\left( k \right)}\left( \boldsymbol{I}-\boldsymbol{D} \right)$ | (S9) |
| --- | --- |

If more than half of the product j is produced in its primary industry, the iterative formula can be further decomposed as shown in Eq. S10.

| ${z_{ij}}^{(k+1)}=u_{ij}-\sum_{\begin{aligned} h=1 \\ h\neq j \end{aligned}}^{n} d_{hj}{z_{ih}}^{\left( k \right)}+\sum_{\begin{aligned} h=1 \\ h\neq j \end{aligned}}^{n} d_{jh}{z_{ij}}^{\left( k \right)}$ | (S10) |
| --- | --- |

According to the above formula, it can be found that the product *i* inputs into the production of the product *j* equals the product *i* inputs into the production of industry *j*, minus the product *i* inputs to the production of another product *h* by industry *j*, plus the product *i* inputs into the production of product *j* by another industry *h*. If the sum of the second and third term is negative and its absolute value exceeds that of the product *i* inputs into the production of industry *j*, this typically arises due to ancillary activities, data heterogeneity, classification differences, or statistical errors, leading to the issue of negative values. To address these negatives, Almon^1^ introduces the scale factor $f_{ij}^{(k)}$ for adjustment, as expressed below.

| $f_{ij}^{(k)}=\left\{\begin{matrix} \frac{u_{ij}}{\sum_{\begin{matrix} h=1 \\ h\neq j \end{matrix}}^{n} d_{jh}z_{ij}^{(k)}}\text{, }u_{ij}<\sum_{\begin{matrix} h=1 \\ h\neq j \end{matrix}}^{n} d_{jh}z_{ij}^{(k)} \\ 1\text{ , }u_{ij}\geq\sum_{\begin{matrix} h=1 \\ h\neq j \end{matrix}}^{n} d_{jh}z_{ij}^{(k)} \end{matrix}\right.$ | (S11) |
| --- | --- |

Here, we can rewrite the iterative formula with the scale factors $f_{ij}^{(k)}$ as Eq. S12.

| ${z_{ij}}^{(k+1)}=u_{ij}-f_{ij}^{(k)}\sum_{\begin{aligned} h=1 \\ h\neq j \end{aligned}}^{n} d_{hj}{z_{ih}}^{\left( k \right)}+\sum_{\begin{aligned} h=1 \\ h\neq j \end{aligned}}^{n} f_{ih}^{(k)}d_{jh}{z_{ij}}^{\left( k \right)}$ | (S12) |
| --- | --- |

Building on this, Almon’s algorithm eliminates the negative values by introducing scale factors that scale down the removal and entry terms of secondary products during the iteration process.

**References**

1. Almon, C. Product-to-product tables via product-technology with no negative flows. *Econ. Syst. Res.* **12**, 27-43 (2000).
2. Eurostat. *Eurostat Manual of Supply, Use and Input-Output Tables* <https://ec.europa.eu/eurostat/web/products-manuals-and-guidelines/-/KS-RA-07-013> (2008).

**Text S2. Derivation of deviations between emissions estimated by product technology assumption and other methods.**

In this section, the product technology assumption (PTA) method forms the basis for deriving the measurement formulas for industry-level emissions and emissions based on the industry technology assumption (ITA). This approach helps to elucidate the mechanism behind the observed deviations between the two types of emissions.

**Deviations between PTA-based product-level and industry-level emissions.**

Based on the Eq. 5 in the main text, industry-level emissions can be expressed in terms of product-level emissions under the PTA method, as shown in Eq. S13:

| $\left[ \begin{matrix} C_{11}^{'} & \cdots& C_{1j}^{'} \\ \vdots& \ddots& \vdots\\ C_{k1}^{'} & \cdots& C_{kj}^{'} \end{matrix} \right]=\left[ \begin{matrix} P_{11} & \cdots& P_{1i} \\ \vdots& \ddots& \vdots\\ P_{k1} & \cdots& P_{ki} \end{matrix} \right]\left[ \begin{matrix} d_{11} & \cdots& d_{1j} \\ \vdots& \ddots& \vdots\\ d_{i1} & \cdots& d_{ij} \end{matrix} \right]$ | (S13) |
| --- | --- |

where $C_{kj}^{'}$ represents the industry-level emissions from the *j*-th industry consuming the *k*-th fuel, expressed in terms of product-level emissions based on PTA. According to Eq. S13, industry-level emissions are allocated from the product-level emissions according to the market share of each product manufactured by industries. This allocation introduces a deviation between the sectoral emissions measured at the industry level and those based on PTA, due to the existence of secondary products. To clarify this deviation mechanism, we expand the expression for $C_{11}^{'}$ as an example.

| $C_{11}^{'}=P_{11}d_{11}+P_{12}d_{21}+\cdots+P_{1i}d_{i1}$ | (S14) |
| --- | --- |

Eq. S14 illustrates the reallocation of product-level emissions through the production in the 1st industry, constituting the industry-level emissions for the 1st industry. The deviation between the industry-level emissions and the product-level emissions based on PTA can be further calculated, as shown in Eq. S15.

| $C_{11}^{'}-P_{11}=P_{11}\left( d_{11}-1 \right)+P_{12}d_{21}+\cdots+P_{1i}d_{i1}$ | (S15) |
| --- | --- |
| $=\sum_{2}^{i} P_{1i}d_{i1}-P_{1}\left( 1-d_{11} \right)$ |  |

In this context, when $d_{11}=1$ and $d_{i1}=\text{0}$ (i.e. the product is produced by only one industry, and the industry produces only one product), the industry-level emissions are equal to the product-level emissions, resulting in zero deviation. However, when $d_{11}\neq\text{1}$, the values of $d_{i1}$ and the disparity between $P_{11}$ and $P_{1i}$ determine the extent of the deviations between the industry-level and product-level emissions.

**Deviations between PTA-based and ITA-based product-level emissions.**

The ITA approach for estimating product-level emissions assumes that all products produced within the same industry share the same input structure. In other words, they are manufactured using identical processes, regardless of the specific product. Consequently, when estimating product-level emissions, all products in the same industry are assigned the same CO_2_ emissions intensity per unit of output. Given known industry-level emissions, the emissions for each industry are allocated according to the proportion of each product produced. Subsequently, the emissions from secondary products are transferred to their respective product sectors, yielding the emissions for each product sector, as shown in Eq. S16 to Eq. S18.

| $t_{ij}=\frac{s_{ij}}{x_{j}^{I}}$ | (S16) |
| --- | --- |
| $\boldsymbol{I}=\boldsymbol{C}\times\boldsymbol{T}^{\boldsymbol{T}}$ | (S17) |
| $\left[ \begin{matrix} I_{11} & \cdots& I_{1i} \\ \vdots& \ddots& \vdots\\ I_{k1} & \cdots& I_{ki} \end{matrix} \right]=\left[ \begin{matrix} C_{11} & \cdots& C_{1j} \\ \vdots& \ddots& \vdots\\ C_{k1} & \cdots& C_{kj} \end{matrix} \right]\left[ \begin{matrix} t_{11} & \cdots& t_{i1} \\ \vdots& \ddots& \vdots\\ t_{1j} & \cdots& t_{ij} \end{matrix} \right]$ | (S18) |

Here, $s_{ij}$ denotes the output of the *i*-th product produced by the *j*-th industry, and $x_{j}^{I}$ denotes the total output of the *j*-th industry, with the origin data derived from the China supply table. $\boldsymbol{T}$ denotes the product mix matrix, with the element $t_{ij}$ representing the fraction of the total output of the *j*-th industry that is in the form of *i*-th product. $\boldsymbol{T}^{\boldsymbol{T}}$ denotes the transpose of matrix $\boldsymbol{T}$. $\boldsymbol{I}$ is the CO_2_ emission matrix for product sectors estimated by ITA, with the element $I_{ki}$ denoting the CO_2_ emission generated by combustion of fossil fuel *k* in the *i*-th product. $\boldsymbol{C}$ represents the CO_2_ emission matrix of industry sectors, with the element $C_{kj}$ denoting the CO_2_ emission generated by burning fuel *k* in the *j*-th industry sector, i.e. the industry-level emissions.

Substituting Eq. 5 into Eq. S18, the expression for sectoral emissions under ITA can be rewritten as follows:

| $\left[ \begin{matrix} I_{11}^{'} & \cdots& I_{1i}^{'} \\ \vdots& \ddots& \vdots\\ I_{k1}^{'} & \cdots& I_{ki}^{'} \end{matrix} \right]=\left[ \begin{matrix} P_{11} & \cdots& P_{1i} \\ \vdots& \ddots& \vdots\\ P_{k1} & \cdots& P_{ki} \end{matrix} \right]\left[ \begin{matrix} d_{11} & \cdots& d_{1j} \\ \vdots& \ddots& \vdots\\ d_{i1} & \cdots& d_{ij} \end{matrix} \right]\left[ \begin{matrix} t_{11} & \cdots& t_{i1} \\ \vdots& \ddots& \vdots\\ t_{1j} & \cdots& t_{ij} \end{matrix} \right]$ | (S19) |
| --- | --- |

where $I_{ki}^{'}$ represents the ITA-based emissions from the *i*-th product consuming the *k*-th fuel, expressed in terms of product-level emissions based on PTA. The matrix $\boldsymbol{G}=\boldsymbol{D}\boldsymbol{T}^{\boldsymbol{T}}$ is defined as the matrix of contributing coefficients for product-level emissions, where the element $g_{ij}$ denotes the contribution of *i*-th product’s emissions, as measured by PTA, to the *j*-th product’s emissions, as estimated by ITA.

| $\boldsymbol{G}=\boldsymbol{D}\boldsymbol{T}^{\boldsymbol{T}}=\left[ \begin{matrix} d_{11} & \cdots& d_{1j} \\ \vdots& \ddots& \vdots\\ d_{i1} & \cdots& d_{ij} \end{matrix} \right]\left[ \begin{matrix} t_{11} & \cdots& t_{i1} \\ \vdots& \ddots& \vdots\\ t_{1j} & \cdots& t_{ij} \end{matrix} \right]=\left[ \begin{matrix} g_{11} & \cdots& g_{1j} \\ \vdots& \ddots& \vdots\\ g_{i1} & \cdots& g_{ij} \end{matrix} \right]$ | (S20) |
| --- | --- |

As shown in Eq. S20, the ITA allocates and transfers the products emissions according to the market share of each product and the product mix in each industry. This process results in deviations from PTA-based emissions due to the presence of secondary products and industries. To better understand the underlying mechanism of these discrepancies, we expand an expression of $I_{11}^{'}$ as an example.

| $\left\{ \begin{aligned} I_{11}^{'}=P_{11}g_{11}+P_{12}g_{21}+\cdots+P_{1i}g_{i1} \\ g_{11}=d_{11}t_{11}+d_{12}t_{12}+\ldots+d_{1j}t_{1j} \\ g_{21}=d_{21}t_{11}+d_{22}t_{12}+\ldots+d_{2j}t_{1j} \\ \cdots\\ g_{i1}=d_{i1}t_{11}+d_{i2}t_{12}+\ldots+d_{ij}t_{1j} \end{aligned} \right.$ | (S21) |
| --- | --- |

Eq. S22 illustrates the process of reallocating emissions for each product based on its market share to obtain industry-level emissions, which are then apportioned according to the production proportion of the 1st product across industries and result in the 1st product’s emissions under ITA. The deviation of the emissions from product-level emissions, as measured by ITA, from those based on PTA is given by Eq. S22:

| $I_{11}^{'}-P_{11}=P_{11}\left( g_{11}-1 \right)+P_{12}g_{21}+\cdots+P_{1i}g_{i1}$ | (S22) |
| --- | --- |
| $=\sum_{2}^{\text{i}} P_{1i}g_{i1}-P_{11}\left( 1-g_{11} \right)$ |  |

when $g_{11}=\text{1}$ (i.e. the industry produces only one product, and the product is produced by only one industry), the sectoral emissions measured by ITA are equal to those measured by PTA, resulting in zero deviation. However, when $g_{11}\neq\text{1}$, the values of $g_{i1}$ and the disparity between $P_{1i}$ and $P_{11}$ determine the extent of deviation between ITA-based emissions and PTA-based emissions. It is important to note that the deviation in sectoral emissions measured by ITA is affected by the market share of each product, the product mix of each industry, and the emissions gap between primary and secondary products. As a result, deviations in ITA-based emissions are often higher than those in industry-level emissions.

**Table S2. Classification matching between fossil fuels and energy sectors.**

| **No.** | **Fuels in China’s Energy Statistics** | **Fuels in this study** | **Energy sectors in IOT 2017, 2018, 2020** | **Energy sectors in IOT 1997, 2002, 2007, 2012** |
| --- | --- | --- | --- | --- |
| 1 | Raw coal | Raw coal | Mining and Washing of Coal | Mining and Washing of Coal |
| 2 | Cleaned coal | Cleaned coal |  |  |
| 3 | Other washed coal | Other washed coal |  |  |
| 4 | Briquettes, Gangue | Briquettes |  |  |
| 5 | Crude oil | Crude oil | Extraction of Petroleum and Natural Gas | Extraction of Petroleum and Natural Gas |
| 6 | Natural gas | Natural gas |  |  |
| 7 | Coke | Coke | Processing of Coking Products | Processing of Petroleum, Coking and Nuclear Fuel |
| 8 | Other coking products | Other coking products |  |  |
| 9 | Gasoline | Gasoline | Processing of Petroleum, and Nuclear Fuel |  |
| 10 | Kerosene | Kerosene |  |  |
| 11 | Diesel oil | Diesel oil |  |  |
| 12 | Fuel oil | Fuel oil |  |  |
| 13 | Refinery Gas | Refinery gas |  |  |
| 14 | Naphtha, Lubricants, Petroleum waxes, White spirit, Bitumen asphalt, Petroleum coke, Other petroleum products | Other petroleum products |  |  |
| 15 | Coke oven gas | Coke oven gas | Production and Supply of Gas | Production and Supply of Gas |
| 16 | Blast furnace gas, Converter gas, Other gas | Other gas |  |  |
| 17 | Liquefied Petroleum Gas (LPG) | LPG |  |  |
| 18 | Liquefied Natural Gas (LNG) | LNG |  |  |
